# An initial loading-dose vitamin D versus placebo after hip fracture surgery: baseline characteristics of a randomized controlled trial (REVITAHIP)

**DOI:** 10.1186/1471-2318-14-101

**Published:** 2014-09-09

**Authors:** Jenson CS Mak, Linda A Klein, Terry Finnegan, Rebecca S Mason, Ian D Cameron

**Affiliations:** 1 Department of Geriatric Medicine, Gosford Hospital, Gosford, NSW, Australia; 2 Rehabilitation Studies Unit, Sydney Medical School, University of Sydney, Sydney, New South Wales, Australia; 3 Office of Medical Education, Sydney Medical School, University of Sydney, Sydney, New South Wales, Australia; 4 Department of Geriatric Medicine, Royal North Shore Hospital, Sydney, NSW, Australia; 5 Department of Physiology, School of Medical Sciences, The University of Sydney, Sydney, New South Wales, Australia

**Keywords:** Fragility fractures, Hip fracture, Vitamin D, Aged care, Metabolic bone disorders, Osteoporosis, Rehabilitation, Trauma surgery

## Abstract

**Background:**

Hypovitaminosis D is particularly common among older people with a proximal femoral (hip) fracture. There are currently no agreed strategies for vitamin D replenishment after hip fracture surgery. The REVITAHIP Study is a multisite, double-blinded randomized-controlled trial investigating the effects of an oral vitamin D loading dose on gait velocity after hip fracture surgery. We describe the baseline characteristics of participants, aiming to document hypovitaminosis D and its associations after hip fracture.

**Methods:**

Participants, over 65, recruited within 7 days following hip fracture surgery from 3 Australia hospitals, were randomly allocated to receive a loading dose of vitamin D3 (250,000IU) or placebo, followed by oral maintenance vitamin D3/calcium (800 IU/500 mg) and the usual hip fracture rehabilitation pathway. Demographic and clinical data were collected, including surgical procedure, pre-fracture functional status, Mini Mental State Examination (MMSE) score, serum 25-hydroxyvitamin D (25-OHD), Verbal Rating Scale (VRS) for pain, grip strength and gait velocity. The associations of baseline 25-OHD levels with demographic and clinical data were assessed using Pearson’s correlation, ANOVA and regression analyses.

**Results:**

Two-hundred-and-eighteen people with hip fracture participated in the study. Mean age was 83.9+/-7.2 years, 77% were women and 82% lived in private homes. Fifty-six percent had a subcapital fracture. Mean comorbidity count was 3.13+/-2.0. Mean MMSE was 26.1+/-3.9. Forty-seven percent of participants had hypovitaminosis D (<50 nmol/L). Multivariate regression models demonstrated higher baseline vitamin D levels were significantly associated with higher premorbid Barthel index scores, lower post-operative VRS pain levels and use of vitamin D.

**Conclusion:**

This study cohort shared similar demographic characteristics and comorbidities with other cohorts of people with hip fracture, with the probable exception of less cognitive impairment. Hypovitaminosis D was not as prevalent as previously documented. Patients taking vitamin D supplements and with higher premorbid Barthel index, reflecting greater independence and activity, tended to have higher 25-OHD levels at baseline. Further, lower VRS pain ratings following surgery were associated with higher vitamin D levels. Such associations will need further investigation to determine causation. (ANZCTR number, ACTRN12610000392066).

**Trial registration:**

The protocol for this study is registered with the Australian New Zealand Clinical Trials Registry ANZCTRN ACTRN12610000392066.

## Background

Hip fractures account for the majority of fracture-related health care expenditure and contribute to substantial mortality in men and women over the age of 60 years [1]. By 2040, an estimated 512, 000 hip fractures will occur in the United States each year at a cost of $16 billion per year [2], and by 2050, an estimated 76.7 billion Euros will be spent on this problem in Europe [3]. One year after fracture, 37.1% of men and 26.4% of women will have died, compared with an expected annual mortality of about 10% in this age group [4]. More than 10% of survivors will be unable to return to their previous residence. Most of the remainder will have some residual pain or disability [5]. As most people do not recover fully from a hip fracture, personal and societal costs are substantial. Following hip fracture, in addition to surgery, there is often the need for rehabilitation, outpatient visits for follow-up treatment, and assistance with activities of daily living at home during the recovery period. Improving functional parameters following a hip fracture has the potential to be of great benefit to older people by reducing disability and enhancing quality of life and could also reduce direct treatment costs and costs of long-term community or residential aged care services. Mobility is the key activity underlying functioning and quality of life [6].

Hypovitaminosis D is commonly associated with hip fracture in older people. It occurs because of multiple factors such as decreased sun exposure with reduced skin production of vitamin D and low dietary D2/D3 intake. Vitamin D replacement has been used successfully to reduce such fractures, as well as falls among older people [7-9]. However, in the absence of preventive treatment, hypovitaminosis D following hip fracture may result in proximal muscle weakness, pain, reduced dynamic balance and performance speed [10], affecting mobilization during the acute postoperative and rehabilitation periods [11-15].

The REVITAHIP study (ACTRN12610000392066) is a multisite, randomized controlled trial examining the effect of an initial loading dose of vitamin D3 (250,000 IU) on rehabilitation outcomes following hip fracture surgery [16]. In this paper, we describe the baseline characteristics of participants enrolled in this study. The main objective is to compare the REVITAHIP cohort to other cohorts of studies in which vitamin D has been used proactively to improve outcomes following hip fracture surgery, as well as with the Australian population survey of hip fractures from the Australian Institute of Health and Welfare (AIWH) [6], with a focus on hypovitaminosis D, cognitive and functional status.

## Methods

### Design

This paper presents a cross sectional analysis of the baseline data from the RCT trial. The RCT methodology is described in more detail in the protocol paper [16]. The Northern Sydney Central Coast Area Health Service (NSCCAHS) Harbour Human Research Ethics Committee (HREC) approved the study protocol (HREC Number 10/HARBR/14) on May 13, 2010.

### Participants

Older people admitted to participating public hospital wards in Sydney, Australia, were invited to participate if they were aged 65 years or over, presenting with a hip fracture requiring surgical treatment and able to provide informed consent, either directly or via the “person responsible.” Other inclusion and exclusion criteria are described in the protocol paper [16]. The Northern Sydney Central Coast Area Health Service Harbour Human Research Ethics Committee (HREC) approved the study protocol (HREC Number 10/ HARBR/14) on May 13, 2010.

### Randomization

After consent and completion of the baseline assessment, participants will be formally entered into the study and randomised to intervention or control groups. Randomization will occur using a computer-generated random number schedule with variable block sizes of 2 to 6. It will be performed centrally by an investigator not involved in recruitment or assessments. To conceal allocation, study staff will ring a central telephone number to be notified to which group the participant has been randomized.

### Intervention

Patients in the treatment group received a loading dose of oral vitamin D3 (5 tablets of 50 000 IU, Calciforte Strong [API Pharmaceuticals, New Zealand], total 250 000 IU), ideally within 96 hours but up to 7 days postsurgery. Patients in the control group received 5 placebo tablets identical in appearance to the active tablets, ideally within 96 hours but up to 7 days postsurgery. Both groups were provided with a twice daily oral calcium–vitamin D combination tablet (calcium 500 mg and vitamin D3 400 IU; Calcia, Nycomed Pharmaceuticals: 2 Lyonpark Road, North Ryde NSW 2113). Participants unable to tolerate the oral tablet formulations will be switched to a daily oral sachet of calcium–vitamin D combination effervescent granules (calcium 1000 mg and vitamin D3 880 IU; Calvid, Pharmaceutical Special Products: 2 Albert Road, Moonah, Tasmania. Australia 7009). Those patients in either group with severe hypovitaminosis D (25OHD <10 nmol/L) were commenced on an oral moderate-dose vitamin D of 2000 units twice a day for 14 days to ensure adequate and timely replacement.

### Baseline outcome measures

Participants were reviewed at recruitment for descriptive information including demographic data (age, sex, place of residence), clinical data (surgical procedure, pre-fracture and post-fracture mobility status, medications, 25-hydroxyvitamin D (25-OHD) using DiaSorin immunoassay with cross-reference to Standard Reference Materials [17,18], serum corrected calcium levels, activities of daily living (ADLs)/functional status using the Modified Barthel Index) [19], quality of life (EQ5D) [20], Mini Mental State Examination (MMSE) [21] and Clock-Drawing Tests [22], grip strength (using the participant’s dominant hand on a JAMAR hand dynamometer with maximum reading on 2 attempts [23]) and gait velocity. Weight was measured on body weight scales, whilst height measurements were taken using a ‘drop down’ tape measure fixed at about 2 metres on a wall (and from a tape measure in the supine position when weight-bearing was not possible). Data were collected for the Functional Comorbidity Index [24] using direct medical file review, and whenever necessary, additional information was sought from participant’s medical records.

The study protocol required that a 25-OHD assay be performed prior to the loading dose. In some cases however, the 25-OHD level had been requested on the same day of the loading dose, but was ultimately taken within 24-48 hours after the loading dose. This occurred for 62 (28.4%) of participants. These 62 participants were excluded from analyses using baseline 25-OHD.

### Statistical analysis

Where appropriate, differences between variables of interest were assessed for statistical significance using the Pearson chi-square test for categorical variables and analysis of variance (ANOVA) for continuous variables. Regression analyses were used to examine the degree of association between baseline vitamin D levels and demographic characteristics, fracture type, surgical procedure, and cognitive status - first at the univariate level, followed by multivariate modelling to determine those variables maintaining significance while controlling for all others. A *p* value less than .05 was considered statistically significant. Data were analyzed using IBM SPSS Version 21.0.

## Results

**Figure 1:**
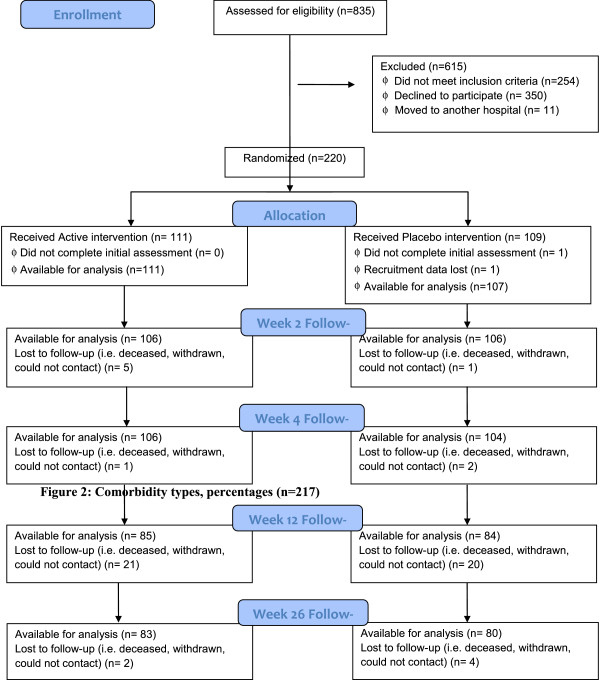
REVITAHIP CONSORT 2014 flow diagram.

**Figure 2:**
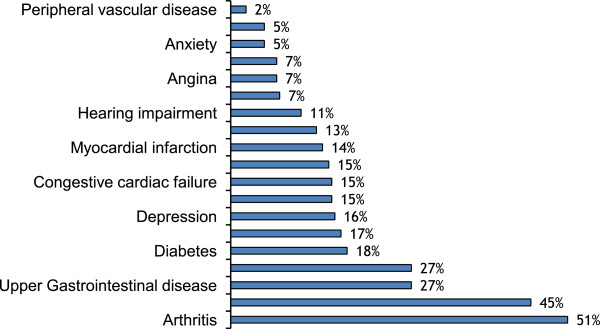
Comorbidity types, percentages (n=217).

A total of 835 suitable patients were identified over the study period (January 2011 to April 2013), of whom 218 consented, were randomized and completed assessments (Figure 1). The participants ranged in age from 65 to 101 years (mean of 83.9+/-7.2 years). A majority (168, 77.1%) were female. Females had a higher mean age than males (84.6 vs 81.6, p < =0.009). Most participants (81.7%) were born in Australia and 93.6% spoke English as the first language. Mean height was 163.0+/-8.7 cm, mean weight was 66.0+/-11.8 kg, and mean BMI was 24.7+/-3.6 m/kg2. Approximately 83% of participants had a pre-injury residence in the community, with 44% of the whole sample living alone. Fourteen percent of participants lived in a residential aged care facility including 4% living in a nursing home. Table 1 summarises the baseline characteristics of the participants.Participants had a mean Functional Comorbidity Index of 3.1+/-2.0, ranging from 0 to 9 separate conditions. The most prevalent comorbid conditions were arthritis and osteoporosis as shown in Figure 2, which provides further details of other comorbid conditions.

**Table 1:** **Baseline characteristics of participants**^**a**^

***Continuous variables***	***Mean +/- SD***
Age in years	83.9+/-7.2
**Categorical variables**	**Percentage**
Gender (% women)	77.2%
Country of birth^c^ (% Australia)	81.4%
Main language spoken^c^ (% English)	93.5%
Body mass index^d^ in kg/m2	24.6+/-3.6
Pre-injury living status^c^ (% at home along)	43.5%
Pre-injury mobility^c^ (% independent)	69.4%
Pre-injury personal ADLs^c^ (% independent)	64.8%
Pre-injury modified Barthel index	87.09+/-21.04
Pre-injury functional comorbidity index	3.12+/-1.95
Comorbidity present:	
Arthritis	49.1%
Osteoporosis	45.4%
Gastrointestinal	27.4%
Visual impairment	27.3%
Hip fracture subtype^e^	- Undisplaced subcapital: 4.3%
	- Displaced subcapital: 46.7%
	- Pertrochanteric simple (2-part): 4.3%
	- Pertrochanteric complex (3-part): 29.0%
	- Intertrochanteric/basicervical: 6.7%
	- Subtrochanteric: 9.0%
Hip fracture surgery^e^	- Cannulated screws: 2.4%
	- Uncemented hemiarthroplasty: 13.7%
	- Cemented hemiarthroplasty: 8.1%
	- Total hip replacement: 18.5%
	- Dynamic hip screw with short plate: 10.4%
	- Dynamic hip screw with long plate: 18.5%
	- Gamma nail: 28.4%
MMSE score (c)	26.0+/-4.35
Using calcium (e)	10.4%
Using vitamin D (e)	10.3%
Using other osteoporosis medications (b)	29.9%
Using psychotropic medication (e)	32.7%
Using antihypertensive medication (e)	54.2%
25-hydroxyvitamin D level (f)	52.7+/-23.5 nmol/L
Serum corrected calcium (e)	2.31+/-0.13 mmol/L
Grip strength (e)	16.1+/-6.8 kgs

^a^All data are expressed in numbers (% of total) except for continuous variables.

^b^Except calcium and vitamin D.

^c^Missing data = 4 (n=216).

^d^Missing data = 28 (n=192).

^e^Missing data = 10 (n=210).

^f^Missing data = 65 (n=155).

The number of medications used prior to hip fracture ranged from 0 to 10 (mean of 4.9+/-2.6). Seventeen percent of participants were either using calcium, vitamin D or both prior to their hip fracture, while 20% were using osteoporosis medications other than calcium or vitamin D, of which 81% were bisphosphonates. Prior to hip fracture 73 (33.5%) participants were using psychotropic medications with 31.5% of these 73 using two or three, including antidepressants (49, 67.1%), anxiolytics/sedatives (16, 21.9%), opioids (15, 20.5%), anticonvulsants (8, 11.0%), and antipsychotics (7, 9.6%).

Mean Modified Barthel Index was 87.1+/-21.0 (range 2-100; median = 100) before hip fracture. This means that, on average, most participants had little or no restrictions in mobility and personal activities of daily living. Specifically, 67.7% of participants reported full independence in their personal hygiene, specifically bathing self (65.4%), feeding self (76.0%), toileting (70.0%), stair climbing (60.4%), dressing (68.7%), bowel control (68.7%), bladder control (62.2%), ambulation (70.0%) and chair to bed transfer (75.1%).

Most (94.5%) participants completed the MMSE (7 were unable and 5 declined). Mean MMSE score was 26.0 +/- 4.3, ranging from 0 to 30, with 16.0% scoring less than or equal to 23 out of 30. In detail, participants were able to correctly answer the following MMSE items: orientation to time (67.8%); orientation to place (76.4%), 3-item register (93.8%), attention (51.4%), 3-item recall (37.0%), naming (82.7%), repeating ‘no ifs ands or buts’ (87.5%), 3-step command (72.1%), read and obey (97.1%), write sentence (89.3%), copy a design (77.2%). Half (50.5%) scored 3 out of 3 in the Clock Drawing Test.

### Hip fracture subtype

In all, 107 (50.5%) patients had a subcapital fracture (9 undisplaced, 98 displaced), while 105 (49.5%) had a trochanteric fracture (10 simple trochanteric, 75 complex pertrochanteric, 20 subtrochanteric). Fracture details for six patients are not known.

### Surgical details and post-operative weight-bearing status

Mean time from fracture to surgery was 41.3+/-32.4 hours (median 36.0) and mean length of surgery was 1.8+/-0.7 hours. Surgical fixation procedures were as follows: gamma nail (29.1%), dynamic/sliding hip screw with long plate (18.3%), total hip replacement (18.3%), dynamic/sliding hip screw with short plate (10.3%), uncemented hemiarthroplasty (eg Austin-Moore) (13.6%), cemented hemiarthroplasty (8.0%) and cannulated screw(s) (2.3%). Details for five patients are not known. At a mean of 3.3 +/- 2.0 days after hip fracture surgery, 73.7% of participants were mobilising with unrestricted weight bearing.

### Function and pain at time of baseline assessment

Following hip fracture, participants had a significantly reduced mean EQ5D Health rating (reflective of function at the time of assessment) with the majority reporting minimal to substantial problems in all functional domains: pain (78.7%), anxiety/depression (26.9%), mobility (96.3%), self-care (94.0%) and usual activity (96.8%). The mean EQ5D health rating was 60.7+/-16.5 (median = 60), ranging from 5 to 100 out of 100. This indicates considerable restriction which is as expected a short time after hip fracture. The mean grip strength was 16.1+/-6.8 kgs at this time, while mean VRS pain was 3.5+/-2.3 (median = 3.0) out of 10. More than half (62.5%) had VRS > =3 and 18.5% had VRS > =5.

### Vitamin D levels

For patients in which 25-OHD levels were assessed prior to the intervention loading dose (n = 156), mean 25-OHD was 52.7+/-23.5 mmol/L (median = 54.0), ranging from 9 to 130 mmol/L. Hypovitaminosis D (<50 nmol/L) was present in 46.8% of these participants. Vitamin D levels classified by groups were noted as follows: 15.4% (<30 nmol/L), 31.4% (30-50 nmol/L), 39.1% (51-75 nmol/L), and 14.1% (>75 nmol/L). Unsurprisingly, participants who had 25-OHD levels assessed after the loading dose was given, showed significantly higher levels than those that were assessed before the loading dose (70.1 vs 52.7 nmol/L, F = 18.4, p < .001), supporting the need for exclusion from the following analyses.

In univariate analyses, variables that were significantly related to pre-loading dose 25-OHD levels were the use of Vitamin D supplements (p = 0.001), use of calcium supplements (p = 0.027), total number of medications used (p = 0.048), total Barthel score as a dichotomous variable (<70/> = 70; p = 0.028) and VRS pain rating (p = 0.028) as shown in Table 2. That is, use of supplements, greater number of medications, higher Barthel score and lower VRS pain ratings were associated with increasing 25-OHD levels. These variables along with those where *p* < 0.20 (i.e. total Barthel score as adults continuous variable, living alone versus with others, and post-operative weight bearing) and with season when 25-OHD measure was taken (which is thought to influence 25-OHD levels though not found to be significant on its own) were examined using multivariate regression modeling. All other demographic and study variables (i.e. age, gender, country of birth, use of psychotropic or other types of medication, total functional comorbidity index, BMI, obesity, EQ5D measures, MMSE total score, fracture type, surgical method, serum calcium levels and grip strength) did not show significant association with 25-OHD levels and were not included in multivariate modeling. The use of Vitamin D supplements, higher total Barthel Index and lower VRS pain maintained significant relationship to increasing 25-OHD levels as shown in the final multivariate model (see Table 3).

**Table 2:** Univariate association between dependent variable 25-hydroxyvitamin D (25O HD) levels before hip fracture surgery (n=155), and baseline characteristics

**Continuous independent variable**^**a**^		**n**	**Correlation coefficient (r)**	**P value**
Number of medications used		156	0.159	0.048*
VRS pain rating		155	-0.177	0.028*
**Categorical independent variable**^**b**^			**Mean (sd)**	**P value**
Living arrangements (living alone/with others)		155		0.095
Using calcium		156		0.027*
	No		51.2 (22.9)	
	Yes		64.2 (25.2)	
Using vitamin D		156		<0.001*
	No		50.3 (22.7)	
	Yes		73.4 (20.1)	

^a^Continuous independent variables were compared individually with 25O HD (continuous dependent variable) using Pearson Correlation Coefficient.

^b^Categorical independent variables were compared individually with 25O HD using ANOVA.

*Indicates statistical significance (p<.05).

**Table 3:** Multivariate regression model: variables associated with baseline 25-hydroxyvitamin D (25-OHD) levels (n= 150) following backward stepwise method

**Variable name**	**B**	**Std. error**	**t**	**p**	**95% confidence interval**
(Constant)	38.553	7.313	5.272	0.000	24.100– 53.006
Using vitamin D (no/yes)	25.633	6.139	4.176	0.000	13.502 – 37.765
Total Barthel -premorbid function(0-100)	0.203	0.079	2.579	0.011	0.048 – 0.359
VRS Pain rating after surgery (0-10)	-1.707	0.739	-2.310	0.022	-3.166 – -0.247

r^2^ = 0.157; model significant at p<0.001.

Variables excluded on 5 previous steps: Using calcium (no/yes), post-operative weight bearing (weight-bearing(WB)/Non-WB+Partial-WB), season of Vit D measurement (winter/other season), pre-admission residence (alone/with others), total number of medications (0-10).

## Discussion

This study has demonstrated that patients with hip fracture are likely to have hypovitaminosis D (46.8%) but a subset was vitamin D replete. The REVITAHIP results for 25-OHD levels were slightly higher than those reported by Ish-Shalom et al [12] but similar to those reported by Papaioannou [13], which ranged from 44.3 to 58.7 nmol/L.

Furthermore, our multivariate model shows that premorbid Barthel index, post-operative VRS pain and prior use of vitamin D together were associated with baseline 25O HD concentrations. This model suggests that patients with lower Barthel index scores and higher reported VRS pain were more likely to have lower 25O HD levels. For example, those with hip fractures who were premorbidly less active with correspondingly lower functional status and those who reported higher pain scores postoperatively were more likely to show hypovitamosis D following their hip fracture operation. Use of Vitamin D preparations prior to fracture had a very clear relationship with higher Vitamin D levels at baseline.

Similar to previous data [5], this study found that hip fracture has significant impact on mobility, personal ADLs and quality of life in the immediate post-acute period. Participants had a significantly reduced mean EQ5D Health rating, with the majority reporting minimal to substantial problems in all functional domains: pain, anxiety/depression, mobility, self-care and usual activity. With regards to comorbidities, we found that hip fractures occurred in older females with multiple comorbidities, particularly arthritis, osteoporosis and visual impairment, which are significantly associated with falls leading to fractures in the elderly. One-sixth of REVITAHIP participants scored below the norm in the MMSE scores, were not in delirium (suggested by the majority being able to complete a 3-step command and were oriented), which suggest the cohort had pre-existing cognitive impairment. However, only 6.9% were documented to have ‘neurological disease’, suggesting that the FCI may have underestimated the prevalence of dementia in our cohort.

A smaller number of REVITAHIP participants (28.9%) were using calcium, vitamin D and/or other osteoporosis medications (e.g. bisphosphonates), despite a much larger number (45%) with previously documented osteoporosis on history, suggesting non-adherence as a contributing factor. Further, those patients with ‘silent osteoporosis’ are likely not being appropriately treated. Furthermore, a third of participants were on psychotropic medications including antipsychotics, opioids, antidepressants, anxiolytics, sedatives and/or anticonvulsants, reflecting the strong role of psychotropic medications as a risk factor for falls leading to hip fracture.

We note that compared to the NICE guidelines [25] which recommended ‘hip fracture surgery (be performed) on the day of, or the day after, admission’ (0-24 hours), our cohort had a longer median time to surgery (36 hours). Most of these delays were attributed to ‘due processes’ and it is possible that, the delay in hip fracture surgery may contribute to a delay in the ‘early mobilisation’ step in patients’ rehabilitation.

The strengths of this study are the inclusion of people with hip fracture who have similar characteristics to the Australian report of 16,518 patients with hip fracture [8] with a similar mean age (83.0 vs 83.9 years), hip fracture subtype (53% vs 51% with neck femur fractures), and mode of surgery (61.3% vs 60.1% internal fixation). The weaknesses of this study are in the under-recruitment of people with severe pre-existing disability compared to the usual population with hip fractures: (1) low number of participants from residential aged care facility; (2) participants with fewer total comorbidities; (3) a small number with preexisting cognitive impairment (16.0%), although one-sixth was actually documented in cognitive impairment in our cohort, suggesting either underreporting of the disease at recruitment, or first diagnosis of disease following a hip fracture. Our cohort of participants had relatively good function with moderate independence prior to their hip fracture, likely due to the inclusion and exclusion criteria. However, the investigators believe that this could also be considered a strength, as it alerts clinicians to potential problems (e.g. in ADLs, mobility, high psychotropic medication use) even in a relatively good functioning cohort. Finally, our participants had a higher level of 25O HD compared to expected, likely owing to a less frail participant population.

## Conclusions

This study cohort shared similar demographic characteristics and comorbidities with other cohorts of people with hip fracture, with the probable exception of less cognitive impairment. Hypovitaminosis D was not as prevalent as documented in some other studies. Patients taking vitamin D supplements and with higher premorbid Barthel index, reflecting greater independence and activity, tended to have higher 25-OHD levels at baseline. Further, lower VRS pain ratings following surgery were associated with higher vitamin D levels, but this is also consistent with greater independence and activity. Such associations will need further investigation to determine causation.

## Competing interests

The authors declared no competing interest with respect to the authorship and/or publication of this article.

## Authors’ contributions

JM participated in conception and design, acquisition of data, analysis and interpretation of data; 2) LK participated in acquisition of data, analysis and interpretation of data; 3) TF, RM & IC participated in conception and design, analysis and interpretation of data. All authors read and approved the final manuscript.

## Pre-publication history

The pre-publication history for this paper can be accessed here:

http://www.biomedcentral.com/1471-2318/14/101/prepub
